# Effectiveness of an educational video as an instrument to refresh and reinforce the learning of a nursing technique: a randomized controlled trial

**DOI:** 10.1007/s40037-012-0013-4

**Published:** 2012-04-28

**Authors:** Loris Salina, Carlo Ruffinengo, Lorenza Garrino, Patrizia Massariello, Lorena Charrier, Barbara Martin, Maria Santina Favale, Valerio Dimonte

**Affiliations:** 1 Human Resource Development, ASOU San Giovanni Battista, Turin, Italy; 2 Department of Public Health and Microbiology, University of Turin Undergraduate Nursing Course, Turin, Italy; 3 Via Torino 20, 10070 Fiano (Turin), Italy; 4 University of Turin Undergraduate Nursing Course, Turin, Italy

**Keywords:** Education, Nursing, Teaching, Methods, Evaluation, Video recording, Video streaming, Learning, Effectiveness

## Abstract

The Undergraduate Nursing Course has been using videos for the past year or so. Videos are used for many different purposes such as during lessons, nurse refresher courses, reinforcement, and sharing and comparison of knowledge with the professional and scientific community. The purpose of this study was to estimate the efficacy of the video (moving an uncooperative patient from the supine to the lateral position) as an instrument to refresh and reinforce nursing techniques. A two-arm randomized controlled trial (RCT) design was chosen: both groups attended lessons in the classroom as well as in the laboratory; a month later while one group received written information as a refresher, the other group watched the video. Both groups were evaluated in a blinded fashion. A total of 223 students agreed to take part in the study. The difference observed between those who had seen the video and those who had read up on the technique turned out to be an average of 6.19 points in favour of the first (*P* < 0.05). The results of the RCT demonstrated that students who had seen the video were better able to apply the technique, resulting in a better performance. The video, therefore, represents an important tool to refresh and reinforce previous learning.

## Introduction

Videos are widely used for supporting and stimulating student comprehension in various contexts: in the classroom, in the laboratory and in distance education [1–3]. Educators understand the benefit of combining auditory and visual enhancement within the traditional lecture. The more technologically advanced European universities have already been making wide use of videos since the 1990s [4–6]. Bradley University, in the USA, has been experimenting extensively with video production. The educational model of this university already foresees a tutoring system with or without face-to-face lessons [7].

Easy access to the web allows the scientific community to acknowledge and promote the exchange of opinions [8]. Skiba describes the transformation of higher education and its impact on nursing education. Nursing education is considered by many to be a pioneer in the use of educational technologies. A challenge is the use of emerging technologies, such as Web 2.0 tools, which will help to bridge the gap between the next generation and faculty in nursing schools. Nurse educators need to understand and use the power of technologies to prepare the next generation of nurses.

Some authors are concerned about the risk that the use of computers will belittle rather than to improve education; moreover, they argue whether the use of technology will ever be able to replace the fundamental role of the teacher in student learning. Despite this, many studies suggest that the use of technology and internet is a useful instrument which favours education [1, 8, 9].

In the literature, various observational studies have demonstrated that the use of video streaming contributes to learning as a powerful instrument for education and for the acquisition of clinical competencies, reducing the gap between theory and practice [10–12]. It has also been highlighted that knowledge is not only acquired through video observation, but as part of the students’ learning process. Moreover, some authors suggest how the use of the video can support students in learning different types of techniques [11, 12]. There are also some observational studies which demonstrate that the introduction of video streaming in schools has been very positive in terms of effectiveness and facilitation of learning. [9, 13–16]. Some authors describe the simulation as a characteristic feature which may render the video particularly effective in stimulating learning and emphasizing the concepts. Watching videos is not a passive but an experiential process [17, 18]. Video use should help students to learn new skills and enhance learning processes in much the same way as experience is acquired during their practical training.

The benefits of using videos are well documented and videos are considered a valid tool for student education [19–22]. Particular reference is given to the pedagogical aspects and the impact of the image, the interaction and integration as key elements of video streaming [23].

Currently, there are few experimental studies which demonstrate video effectiveness applied to traditional nursing techniques [24–26]. Studies carried out in critical care have demonstrated how teaching video use in the emergency situation can enhance performance quality. Einspruch [27] adopted this method with laypersons by estimating the efficacy in a randomized controlled trial (RCT). In an RCT, conducted by medical students, it was demonstrated that a 3 min video play produced a comparable or better performance than that used in the American Heart Association (AHA) course [28]. Cofield [29] carried out an experimental study measuring qualitative variables, suggesting the effectiveness of video streaming to reinforce and enhance learning.

Indications of better video use have been analyzed by various experts, in particular by Race and Shephard [1–3]. Two key elements should prevail: keeping to the content and guaranteeing maximum interactivity. Technological progress and the use of streaming videos has virtually erased problems. Interactivity is, therefore, guaranteed by enabling change in real time of any sequence of the video [27].

The advantage of using YouTube clearly emerges as an instrument for sharing videos and allowing students to participate actively in the discussion on films. YouTube has grown during the last few years and is visited by almost 70 million persons a day. With their free access, the main search engines worldwide (Google, Yahoo) allow comparisons among the entire professional and scientific community, thus offering the possibility to comment and make suggestions, and for free and constructive debate, therefore becoming a reliable and updated point of reference. It is a powerful instrument which makes sharing and exchanging ideas possible [30].

Videos are surely among the most interesting technologies, adding value to learning content. Not only should the image be detailed, but it should offer students the possibility to use more channels, thus enhancing and facilitating learning processes.

However, there is little evidence on the efficacy of video streaming to support student learning. The aim of this study is to verify the effectiveness of video streaming as an instrument for refreshing and reinforcing nursing education.

## Methods

### Design of the study

A two-arm RCT design was chosen and performed in December 2009 at the Undergraduate Nursing Course at Turin University. This is a two-group design: subjects were randomly assigned to the experimental and control groups. Both groups attended lessons in the classroom as well as in the laboratory; a month later the control group received written information to refresh the technique, while the experimental group saw the video. Both groups were given ten minutes to allow them to freshen up on the technique. Students were assessed individually by a blinded evaluator using observational rating scales.

### Participants

The population consisted of 250 undergraduate students attending the nursing course at Turin University.

Students who consented to participate in the study were fully informed about their right to withdraw at any time and the fact that data were to undergo aggregate processing and would be stored safely.

### Randomization

Participants were randomized by a computer-generated randomization scheme and the identification number from the randomized allocation schedule. Students were not told whether they would be seeing the video or reading up on the technique. To guarantee the effective homogeneity between the two groups, data variables influencing student performance, such as age, sex, qualifications, laboratory participation, reading information concerning the technique and development of a working activity in the field, were collected.

### The video

The video chosen involves the technique dealing with ‘moving an uncooperative patient from the supine to the lateral position’. The health care professional uses a sliding sheet to move a mobility-impaired person. The written information was compared with the video, which turned out to be identical regarding both the words and the sequence. The duration of the video was 7.32 min.

### Instrument of appraisal

The appraisal instrument was an observational grid developed to assess student performance. The instrument was a 33-item observational form with the possibility to mark whether a single action had been carried out or not. Each item expresses single actions which together make up the technique (e.g. inform the patient about what is going to happen/wrap the cloth around both shoulders/lift the edge of the bed…). Less important items were given 1 point whereas core items were given 2 points. The assessment forms were anonymous and were identified through a code assigned at the time of enrolment.

Performance was evaluated based on the weighted sum of all the actions making up the procedure.

### Pilot study and evaluation of the sample size

A pilot study was carried out in May 2009 on 21 students with the aim of identifying potential shortcomings, testing the appraisal instrument for the collection of information and assessing the sample size necessary to perform the RCT.

Considering the measuring instrument could supply a range of values between 0 and 53 and taking 5 as the minimum significant value which we would observe, we needed to enrol 100 students for each arm of the study for an 80 % statistical power.

### Statistical analysis

The items that made up the technique were treated as qualitative variables: actions not performed vs actions done correctly. In comparing the experimental and control groups for qualitative variables, the Pearson’s Chi-square or the Fisher’s exact test were used: in particular, the latter was used if at least one cell showed less than five expected values. The Shapiro–Wilk test was performed to assess the normal distribution of quantitative variables: the Student’s *t* test was used for variables normally distributed, while the Mann–Whitney test was used if the variable showed a non-normal distribution (Shapiro–Wilk test statistically significant). All tests were conducted at a significance level of 5 %. The data collected were placed on Excel spreadsheets and analyzed with the Stata 9 statistical programme.

## Results

Altogether, 223 students joined the study: 74 males and 129 females. They were randomized into the experimental group (watching video, *n* = 112) and the control group (written information, *n* = 111).

Table 1 illustrates the characteristics of the study sample (experimental group vs. control group) for some variables: gender, age, whether the participant already had a degree, or was already a certified nursing assistant (CNA). The two groups were homogenous for all these variables so that we can presume that possible differences in their performance are attributable to the use of the video.

**Table 1:** Characteristics of the study sample: experimental group vs. control group

	Experimental group (*n* = 112)	Control group (*n* = 111)	*P*
Sex (M/F)	40/72	34/77	N.s.^
Age (mean ± SD)	22.25 ± 5.9	21.92 ± 4.55	N.s.^§^
Other degree (yes/no)	9/103	12/99	N.s.^
CNA (yes/no)	3/109	2/109	N.s.°

N.s. (*P* > 0.05)

*CNA* certified nursing assistant

^ Pearson’s Chi-square test

° Fisher’s exact test

^§^ Student’s *t* test

The results of the study are shown in Table 2.

**Table 2:** Overall performance: experimental comparison group vs. control group

	Experimental group (*n* = 112)	Control group (*n* = 111)	Mean difference ± SE (CI 95 %)	P*
All items^a^	42.95 ± 6.7	36.76 ± 9.2	6.19 ± 1.08 (4.06–8.32)	0.00001
Minimum score	22	9		
Maximum score	53	53		

*CI* confidence interval, *SE* standard error

* Mann–Whitney test

^a^Data are presented as mean ± SD unless otherwise stated

Table 2 illustrates, relatively to the two groups (experimental and control), the results of the performance. The mean difference observed between those participants who had seen the video and those who had read about the technique was 6.19 points (±1.08 standard error (SE).

The difference between the two groups was statistically significant (*P* < 0.05), so viewing the video led to a better performance in comparison with simply reading up on the technique.

## Discussion

What emerges according to the data we obtained is that videos are beginning to draw serious attention to their learning potential. The result of this study is in conformity with the other experimental studies present in the literature [24–26]. We also demonstrated that those who saw the video were better able to apply the technique. The video lasts 7.32 min. The technical aspect of video duration conforms with what emerged from the literature, which recommends that videos should last no longer than 15 min [22].

The possibility of interrupting the film and seeing some sections again offers the advantage of maintaining a high attention level. However, this means that the user should be able to easily access and view the contents. Therefore, it is necessary for users to simply surf the Net, review, observe and retrieve the material of their choice. As described in the literature, and in particular by Bennet [21], students reported that the use of the video seemed a more effective learning tool since they could easily remember what they saw on the film; moreover, they had the possibility to stop, start and rewind the video to address their specific needs.

Various descriptive studies and opinions of experts assert that the video represents an important opportunity for the student to increase and support learning, thus contributing to a better performance of the technique. But the video cannot completely replace face-to-face lessons, although it widely contributes to and complements education [13]. The video library will be available in additional languages, to allow comparison among the scientific and professional community all over the world. Moreover, it would be of support to those students who are not Italian. The educational use of the video surely offers remarkable advantages for online learning even if it clashes with the limits imposed by the web (the possibility of quickly linking to the web). Furthermore, even if effective, the video has to be part of an educational plan correlated with precise objectives which are reflection, verification, understanding and clarification.

## Conclusions

During the last few years, university education has undergone profound changes in the organisation and methodologies used. Video use and video streaming represent one of these instruments. What emerges from the results of this study is that videos are useful tools for refreshing and reinforcing concepts learnt during nursing courses. The use of educational videos could become a routine instrument for student training. A limitation of the research is the possibility that respondents in the intervention group sought more information on their own. A second limitation is that the difference between the two groups could be a short-term effect, because the refresher training was immediately followed up by the assessment. Follow-up research could explore the permanence of the effect of using video as a tool for refresher training.

## Essentials

- The aim of the study is to verify the effectiveness of the video as an instrument for refreshing and reinforcing nursing education.
- The University’s need to provide a useful tool for students.
- The use of the video contributes to refresher training as a powerful instrument for the education and the acquisition of clinical competencies,
- The use of educational videos could become a routine instrument for student training.
